# The Curcumin Derivative, H10, Suppresses Hormone-Dependent Prostate Cancer by Inhibiting 17β-Hydroxysteroid Dehydrogenase Type 3

**DOI:** 10.3389/fphar.2020.00637

**Published:** 2020-05-08

**Authors:** Yating Cheng, Yan Yang, Yinan Wu, Wencheng Wang, Lichun Xiao, Yifan Zhang, Jianzhong Tang, Ya-Dong Huang, Shu Zhang, Qi Xiang

**Affiliations:** ^1^ Institute of Biomedicine and Guangdong Provincial Key Laboratory of Bioengineering Medicine, Jinan University, Guangzhou, China; ^2^ Biopharmaceutical R&D Center of Jinan University, Guangzhou, China; ^3^ Institute of Materia Medica and Guangdong Provincial Key Laboratory of New Pharmaceutical Dosage Form, Guangdong Pharmaceutical University, Guangzhou, China

**Keywords:** curcumin derivatives, 17β-hydroxysteroid dehydrogenase type 3, inhibitor, prostate cancer, hormone-dependent

## Abstract

The 17β-hydroxysteroid dehydrogenase type 3 (17β-HSD3) enzyme is a potential therapeutic target for hormone-dependent prostate cancer, as it is the key enzyme in the last step of testosterone (T) biosynthesis. A curcumin analog, H10, was optimized for inhibiting T production in LC540 cells that stably overexpressed 17β-HSD3 enzyme (LC540 [17β-HSD3]) (P < 0.01), without affecting progesterone (P) synthesis. H10 downregulated the production of T in the microsomal fraction of rat testes containing the 17β-HSD3 enzyme from 100 to 78.41 ± 7.41%, 51.86 ± 10.03%, and 45.14 ± 8.49% at doses of 10, 20, and 40 μM, respectively. There were no significant differences among the groups with respect to the protein expression levels of 17β-HSD3, 3βHSD1, CYP17a1, CYP11a1, and STAR, which participate in 17β-HSD3-mediated conversion of androgens to T (P > 0.05). This indicated that H10 only inhibited the enzymatic activity of 17β-HSD3 *in vitro*. Furthermore, H10 inhibited the adione-stimulated growth of xenografts established from LNCaP cells in nude mice *in vivo*. We conclude that H10 could serve as an effective inhibitor of 17β-HSD3, which in turn would inhibit the biosynthesis of androgens and progression of prostate cancer.

## Introduction

Prostate cancer is not only the second most commonly diagnosed cancer, but also the sixth leading cause of cancer-related deaths among men, worldwide (Morote et al., 2016). The rate of incidence of prostate cancer is increasing in Asian populations (Teoh et al., 2019). The androgens, namely, testosterone (T) and dihydrotestosterone (DHT), play important roles in the development, growth, and progression of hormone-dependent prostate cancer.

At present, there are multiple treatment strategies for hormone-dependent prostate cancer, including orchidectomy and the administration of androgen receptor (AR) blockers, luteinizing hormone-releasing hormone (LHRH) agonists, and 5α-reductase inhibitors (Estévez et al., 2016; Fang and Zhou, 2019). However, the widely used chemical castration strategy with the depot LHRH agonist failed to achieve castration levels for T in nearly 20% of men (Sim et al., 2019). Despite the impressive clinical activity of the second-generation antiandrogens, including enzalutamide and ARN-509, acquired resistance invariably develops in patients with prostate cancer (Rice et al., 2019). However, enzalutamide was associated with increasing the risk of fatigue and cardiovascular events (Roviello et al., 2016). Hormonal treatments acting upstream of the reactions catalyzed by the 17β-hydroxysteroid dehydrogenase type 3 (17β-HSD3) enzyme can affect the systemic balance of other hormones, resulting in undesirable adverse effects on sexual interest and function, as well as on bone mineral density (Yu et al., 2015). Therefore, the identification of novel therapeutic targets and drugs for the treatment of hormone-dependent prostate cancer has become a popular topic of research in the field of medicine.

**Figure 1:**
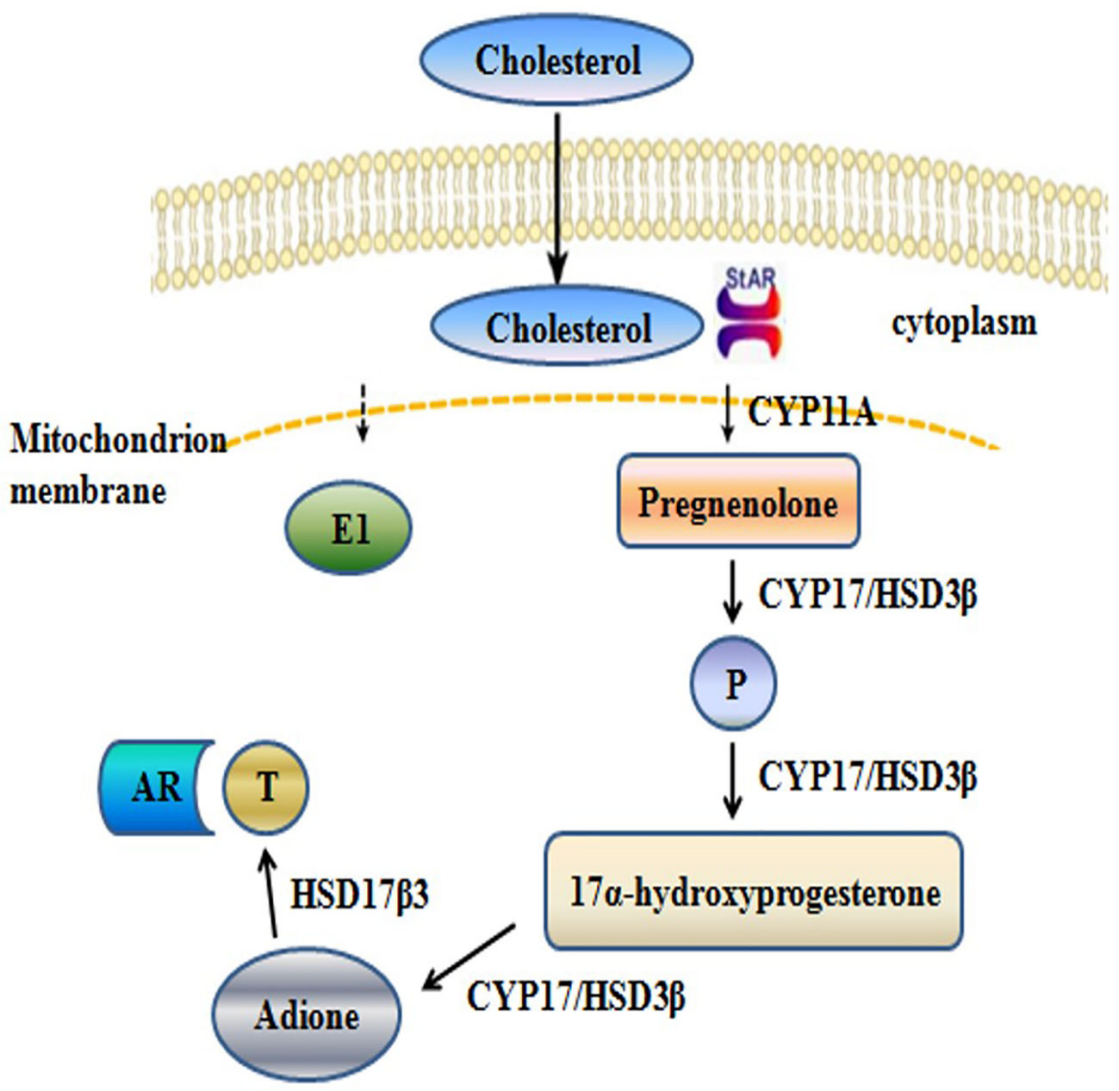
Schematic depicting the synthesis of T.

The synthesis of T involves a series of enzymatic reactions catalyzed by five proteases, including CYP11A, CYP17, 3β-HSD1, 17β-HSD3, and STAR (**Figure 1**). In the last step of T synthesis, a key enzyme, 17β-HSD3, converts androstenedione to active circulating T in the presence of nicotinamide adenine dinucleotide phosphate (NADPH) (Djigoué et al., 2015; Ning et al., 2017). The 17β-HSD3 enzyme catalyzes the biosynthesis of approximately 50% of the total amount of androgen in men (Mendonca et al., 2017). Therefore, 17β-HSD3 has been recognized as a promising therapeutic target for reducing the levels of the circulating androgens and suppressing the proliferation of androgen-sensitive tumors (Kenmogne et al., 2017). The inhibition of 17β-HSD3 might provide an effective treatment strategy for hormone-dependent prostate cancer. Moreover, treatment with specific 17β-HSD3 inhibitors may result in fewer off-target effects, compared with those following treatment with AR antagonists. The upregulation of 17β-HSD3 in prostatic tumors induces the accumulation of 17β-HSD3 inhibitors in the tumor tissues rather than in the normal tissues (Cortés-Benítez et al., 2019).

Numerous studies have investigated the potency of 17β-HSD3 inhibitors in the last decade (Kenmogne et al., 2016; Bacsa et al., 2017; Pourbasheer et al., 2017; Yang et al., 2017; Yazawa et al., 2019), however, none of these inhibitors are currently under investigation in clinical trials (Barbosa et al., 2015). Inhibitors of 17β-HSD3 can either work at the gene level, targeting the mRNA expression of the 17β-HSD3 gene, or inhibit the activity of the enzyme. Curcumin, extracted from *Rhizoma zedoariae* and *turmeric rhizom*e, has diverse pharmacological activities, including anti-inflammatory, anti-tumor, and antioxidant properties (Yang et al., 2015; Ganjali et al., 2017). Curcumin is being studied in clinical trials, primarily for the treatment of diseases related to cancer (Kunnumakkara et al., 2017). The 3,5-bis(benzylidene)-4-piperidone series of curcumin analogues, differing with respect to the substitutions on the two aromatic rings. It has been demonstrated that the presence of electron-withdrawing substituents in the aromatic rings enhances their anti-tumor activity, while a para-substitution with an electron-donating group diminishes their anti-proliferative activity (Awasthi et al., 2016). For instance, chalcones carrying electron withdrawing groups in positions 2 and 6 of the aromatic rings have been reported to act as potent inhibitors of endothelial cell proliferation (Nuti et al., 2017). Besides, the 3,5-bis(benzylidene)-4-piperidones with electron-withdrawing (3,4-(OH)(2)) substitutions in the benzene ring may possess good antioxidant activities.

In an earlier study, we synthesized 12 mono-carbonyl analogs of curcumin that showed inhibitory activity against 17β-HSD3 (Yuan et al., 2014). Among these, compound H10, carrying two chloride substituents in the aromatic rings, was screened and selected due to its 17β-HSD3 inhibitory potential. H10 repressed the production of T, but did not alter the expression of 17β-HSD3. In this study, we identified and characterized H10 as an inhibitor of 17β-HSD3, and aimed to investigate its efficacy in the treatment of androgen-dependent prostate cancer using *in vitro* and *in vivo* studies.

## Materials and Method

### Materials

The candidate 17β-HSD3 inhibitors, of purity greater than 95%, were synthesized by Dr. Jiyan Pang of Sun Yat-sen University. LC540 cells (ATCC^®^ CCL-43™) were purchased from Bioleaf Biotech Co., Ltd, Shanghai, China. The LC540 cells stably overexpressed 17β-HSD3 (LC540 [17β-HSD3]), and were handled by Dr. Yan Yang. LNCap (ATCC^®^ CRL-1740™) was purchased from Shanghai Institutes for Biological Sciences (Shanghai, China).

Male Sprague-Dawley (SD) rats, aged 5–8 weeks, and weighing 200 ± 20 g (animal qualified certificate No. 44007200061277) and 5-week old male nude mice, weighing 20 ± 2 g (animal qualified certificate No. 44007200068302) were purchased from the animal center of Guangdong Province. The animals were individually housed in different rooms at a constant temperature of 25 ± 2°C and a relative humidity of 55 ± 10%, under a 12-h light/dark cycle, and were allowed *ad libitum* access to food and water. The experimental protocol adopted in this study was approved by the Ethics Review Committee for Animal Experimentation of Jinan University (ethical review No. 20170301003), and all the experiments were conducted in accordance with the Guide for the Care and Use of Laboratory Animals by the National Institutes of Health (NIH Publication No. 8023, revised 1996).

### *In Vitro* Model for Screening 17β-HSD3 Inhibitors

The 17β-HSD3 cDNAs were obtained by reverse transcription (RT-PCR) using the total mRNA derived from the testes, with the lentiviral pLVX-EF1α-IRES-Zs Green1 Vector (Clonetech, USA). In order to produce recombinant lentiviruses, the plasmid DNA was transfected into 293T cells. The lentivirus pellets containing the 17β-HSD3 cDNAs were collected after 48, 60, and 72 h of transfection. The supernatants were filtered through a 0.45 μm filter. The LC540 cells were cultured in a 24-well plate at a density of 2 × 10^5^ cells/well, and the medium was subsequently replaced by 2 ml of fresh medium containing the viral pellets and 6 μg/ml of polybrene. After 12 h, the medium was replaced with fresh medium. In order to screen the stably transfected cells, the transfected cells were grown in a medium containing 500 μg/ml of geneticin (G418). The medium was replaced every 2–3 days. The integration and expression of 17β-HSD3 was confirmed by RT-PCR. The LC540 (17β-HSD3) cells were incubated at ∼80% confluency with the candidate compounds in 12-well cell culture plates. After 24 h of treatment, the cells were collected for analyzing the production of T and progesterone (P).

### T and P Content Assay

The content of T and P were detected using the Iodine [^125^I] Testosterone Radioimmunoassay Kit, Iodine [^125^I] Progesterone Radioimmunoassay Kit, and Androstenedione Radioimmunoassay Kit (Beijing North Institute of Biotechnology Co., Ltd), according to the manufacturer’s instructions.

### Detection of 17β-HSD3 mRNA Levels in LC540 (17β-HSD3) Cells by RT-qPCR

The curcumin analog, H10, was selected due to its 17β-HSD3 inhibitory activity, and its effects on the levels of 17β-HSD3 mRNA in LC540 (17β-HSD3) cells were further analyzed. Briefly, the LC540 (17β-HSD3) cells were seeded in Dulbecco’s Modified Eagle Medium (DMEM) supplemented with 10% fetal bovine serum, 100 IU/ml penicillin, and 100 μg/ml streptomycin at a density of 200,000 cells/well in a 12-well plate (BD Falcon) at 37°C in a humidified atmosphere of 5% CO_2_. The experiment was performed in triplicate using different concentrations of H10 over a duration of 24 h. The mRNA was purified using the HiPure Total RNA Kits. A 2 μg aliquot of each mRNA sample was used to generate the cDNA, with the iScript™ cDNA Synthesis Kit. RT-PCR was performed with a Rotor Gene 2000 Real-Time Cycler using 1 μl of cDNA in Taqman universal PCR master mix and Taqman expression assays containing probes and primers for 17β-HSD3. The following probes and primers were used for the PCR: F: *5′-*AACAGTTCCTCCTTTCCGTG-3*′* and R: *5′-*AATGAATAGGCTTT CCCGAT-3*′*, or RPS 16: F: *5′-*AAGTCTTCGGACGCAAGAAA-3*′* and R: *5′-*TTGCCCA GAAGCAGAACAG-3*′*. The conditions of RT-qPCR were as follows: the mixture was initially kept at 95°C for 10 min, followed by 40 cycles of holding at 95°C for 15 s, and subsequently at 60°C for 60 s. The relative mRNA expression was calculated using the comparative quantification algorithm in Rotor Gene 6 Software.

### Enzyme Activity Assay With Microsomal Fraction of 17β-HSD3 From Rat Testes

The influence of H10 on the activity of the 17β-HSD3 enzyme was subsequently investigated. The microsomes from the testes of rats were collected and used to examine the enzymatic activity of 17β-HSD3. Briefly, the testes were homogenized in cold 0.01 mM PBS buffer and centrifuged at 700 × g for 30 min. The supernatants were transferred to fresh tubes and centrifuged at 10,000 × g for another 30 min. The resulting supernatants were centrifuged at 105,000 × g for 1 h. The pellets were resuspended and the concentration of the proteins were measured using the Micro BCA™ Protein Assay Kit (Thermo Scientific), according to the manufacturer’s instructions.

In order to determine the effect of H10 on the enzymatic activity of 17β-HSD3, the microsomes, containing 0.1 μM androstendione (Mich Scientific) and 0.2 mM NADPH (KeyGEN BioTECH), were treated with H10 at varying concentrations for 60 min at 37°C. The total reaction volume was maintained at 1 ml. The reactions were subsequently ceased by the addition of 2 ml of ice-cold ether, following which the ether was removed by passing a stream of nitrogen gas. The concentration of T that was produced was determined by the Immunoassay System (Beckman UniCel DxI 800).

### Western Blot

The LC540 (17β-HSD3) cells were treated with H10 at different concentrations of 0.25, 0.5, and 1 μM for 48 h and were subsequently collected for western blotting. The cells were lysed in radio immunoprecipitation assay buffer (Cell Signaling Technology, Beverly, MA, USA) on ice for 30 min and subsequently centrifuged at 12,000 rpm for 30 min at 4°C. The supernatants were collected, and the protein concentrations were measured with a Bicinchoninic Acid Protein Assay Kit (Life Technologies, Carlsbad, CA, USA), according to the manufacturer’s instructions. Sodium dodecyl sulfate polyacrylamide gel electrophoresis and immunoblotting were performed according to the standard protocols and visualized using the ChemiDoc Imaging System (UVP, Upland, MA, USA). Antibodies against CYP11, CYP17, 3β-HSD1, 17β-HSD3, STAR, and GAPDH (Anity Biosciences, Cincinnati, OH, USA, 1:1,000), and an HRP-conjugated secondary antibody (Cell Signaling Technology, Boston, MA, USA, 1:5,000) were used.

### H10 Reduced the Serum Levels of T in Male SD Rats

Male SD rats (200 ± 20 g) were randomly divided into five groups (n = 6), and subsequently weighed and marked. The rats in Group A were subjected to castration surgery (CTX) 7 days prior to the administration of H10. On the other hand, the complete reproductive function of the animals in Groups B, C, D, and E were kept intact (INT). The animals in Group A and Group B received intraperitoneal (i.p.) injections of 1 ml solvent comprising 10% DMSO:90% aqueous solution of methyl cellulose (0.4%, w/v), on a daily basis for 1 week. The animals in Groups C, D, and E received i.p. injections of 1 ml solution of H10 at doses of 10, 30, and 50 mg/kg, respectively, every morning on and empty stomach for one week. On the 7^th^ day, blood was collected from the posterior orbital venous plexus 6 h after the last administration. The rats were then humanely euthanized, following which the prostate, seminal vesicles, and testes were dissected and weighed. The blood samples were kept on ice, and subsequently centrifuged at 4,500 rpm for 5 min at 4°C. The serum was collected and stored at −20°C until further analyses. The levels of T and P in the samples were detected using the Iodine [^125^I] Testosterone Radioimmunoassay Kit and the Iodine [^125^I] Progesterone Radioimmunoassay Kit, respectively.

### *In Vivo* Inhibition of Adione-Stimulated Proliferation of LNCaP Tumor in Nude Mice

Nude male mice received an i.p. injection of androstenedione 24 h prior to receiving the tumor xenograft. The mice were subcutaneously inoculated with 1 × 10^7^ LNcap cells in 200 μl Matrigel^®^ (BD Biosciences, Franklin Lakes, NJ, USA; #356234) into the right flank, following which the mice received i.p. injections of androstenedione on every alternate day (**Figure 5C**). When the tumor volume reached approximately 100 mm^3^, the mice were randomly divided into five groups (n = 4) and were separately administered i.p. injections of 0.2 ml of the substances mentioned hereafter on every alternate day. The five groups separately received 0.2 ml of either normal saline (NS), or a solvent comprising 10% DMSO:90% methylcellulose (0.4%, w/v), or 10 mg/kg of H10, or 30 mg/kg of H10, or 50 mg/kg of H10. The tumor volumes were measured and calculated by the following formula: volume = 0.52 × length × width^2^ (Pascal et al., 2014). The body weight and tumor volumes were measured once on every alternate day. After 4 h of administration, the plasma samples were collected from the eye sockets of the nude mice prior to sacrifice, on the 14^th^ day. The tumors were collected, weighed, and frozen in liquid nitrogen. The levels of T in the serum and the tumor supernatants were measured for each group with a radioimmunoassay kit.

### Histological Examination

For histological analyses, the tumors were initially fixed in 4% neutral buffered paraformaldehyde, and sequentially dehydrated in an ethanol series with increasing concentration gradients, and subsequently cleared by xylene. The tissue samples were then incubated overnight in a solution of paraffin and xylene in an oven for removing the xylene, following which the tissues were embedded in paraffin. The paraffin-embedded samples were sectioned into 5.0 μm-thick sections for fixing onto the glass slides. Following dewaxing and hydration, the sections were stained with H&E for microscopic investigation (Olympus IX71, Tokyo, Japan).

### Immunohistochemistry

The tumors were fixed in formalin. The paraffin-embedded tissue blocks were dewaxed, rehydrated, and blocked for studying the endogenous peroxidase activity. Antigen retrieval was performed in a sodium citrate buffer (0.01 mol/L, pH 6.0) in a microwave oven at 1,000 W for 10 min. Nonspecific antibody binding was blocked by incubating with 5% bovine serum albumin in PBS for 30 min at room temperature. The slides were then individually incubated with anti-Ki-67 (at 1:100; Affinity), anti-CD-31 (at 1:100; Affinity), anti-AR (at 1:100; Affinity), and anti-17β-HSD3 (at 1:100; Affinity) antibodies at 4°C overnight. After rinsing with PBS, the slides were washed and incubated with rabbit secondary antibodies for 40 min. After washing four times with PBS, the sections were treated with DAB and hematoxylin for staining and re-dying the nucleus, respectively. The sections were finally dehydrated and sealed with Permount™ Mounting Medium for microscopic observation (Olympus IX71, Tokyo, Japan).

### Statistical Analyses

All the data are represented as the mean ± standard deviation (sd). The experiments were repeated at least three times. The data obtained from the different groups were compared by one-way analysis of variance followed by Tukey’s test, using GraphPad Prism 6 software (GraphPad, Inc., La Jolla, CA, USA). *P* values <0.05 were considered to be statistically significant.

## Results

### *In Vitro* Analysis of 17β-HSD3 Inhibitors

**Figure 2:**
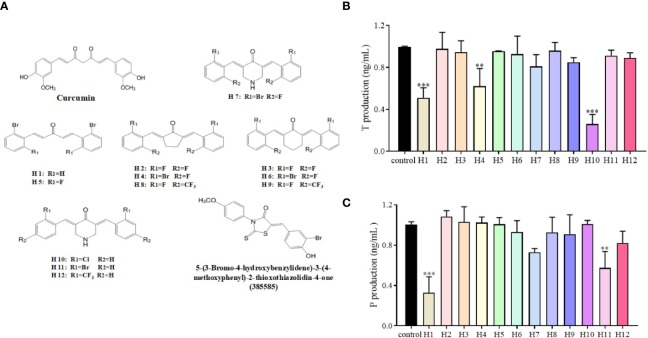
Effects of compounds H1-H12 on LC540 cells transfected with 17β-HSD3. **(A)** The chemical structures of curcumin and its derivatives, as well as of 5-(3-bromo-4-hydroxybenzylidene)-3-(4-methoxyphenyl)-2-thioxothiazolidin-4-one (385585). Assays for **(B)** T and **(C)** P for assessing the effects of H1-H12 on LC540 cells transfected with 17β-HSD3. The results represent the mean ± standard deviation (sd, n = 3); **P < 0.01, and ***P < 0.001 are considered significant *vs.* the control.

The LC540 cells that had been transfected with a cDNA vector coding for 17β-HSD3 (LC540 [17β-HSD3]) were used to screen the 17β-HSD3 inhibitors. The inhibition of 17β-HSD3 suppresses the production of T but does not affect the production of P. Twelve mono-carbonyl analogs of curcumin were synthesized as candidate 17β-HSD3 inhibitors. The LC540 (17β-HSD3) cells were treated with the candidates compound for 24 h. The production of T was subsequently measured. The results demonstrated that compound H10, having two chloride substituted aromatic rings, significantly inhibited the production of T in a dose-dependent manner (**Figure 2**). Therefore, compound H10 was selected for further studies.

### H10 Decreased the Production of T and P but Did Not Affect the Expression of 17β-HSD3 mRNA and Protein

**Figure 3:**
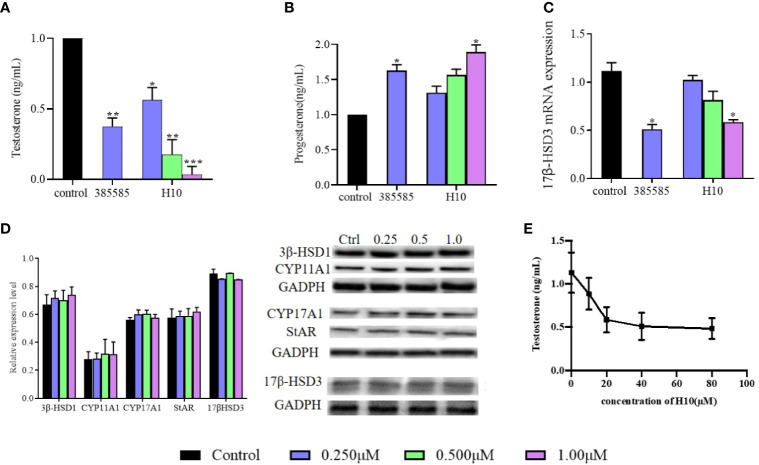
Effects of H10 on 17β-HSD3 in LC540 (17β-HSD3) cells. Effects of H10 on the production of **(A)** T and **(B)** P. **(C)** The expression of 17β-HSD3 mRNA. **(D)** The expression levels of the proteins in the T synthesis pathway were detected by western blotting following treatment with H10. The concentrations of H10 were 0.25, 0.5, and 1 μM, while the concentration of 3858858 (positive control) was 0.25 μM. **(E)** T produced from the microsomal fraction of rat testes containing 17β-HSD3. The data are reported as the mean ± sd (n = 3) of results obtained from individual experiments. *P < 0.05, **P < 0.01, and ***P < 0.001 are considered to be significant, *vs.* the control.

The relationship between the inhibitory effects of H10 and the expression of 17β-HSD3 mRNA and protein were further investigated. The expression of 17β-HSD3 mRNA and protein were examined, and a known 17β-HSD3 inhibitor, 385585 (Merck KGaA, Darmstadt, Germany; #D00130826), was used as the positive control. As depicted in **Figure 3A**, the reduction in the production of T by H10 was significantly higher than that by 385585 and that of the normal control group. H10 inhibited the production of T in a dose-dependent manner, however, there was no significant difference in the levels of P at different doses (*P* > 0.05, **Figure 3B**). The results of RT-qPCR (**Figure 3C**) demonstrated that H10 did not affect the mRNA expression of 17β-HSD3 in LC540 (17β-HSD3) cells, although 0.25 μM of 385585 decreased the expression of 17β-HSD3 mRNA to 54.5 ± 15%.

The expression levels of the proteins were evaluated by western blotting. The LC540 (17β-HSD3) cells were treated with H10 at three different concentrations, 0.25, 0.5, and 1 μM, for 48 h. The results revealed that there were no significant differences in the levels of 17β-HSD3, CYP11A, CYP17, 3β-HSD1, and STAR following treatment with H10 at different concentrations (*P* > 0.05, *vs* the control group, **Figure 3D**).

### H10 Inhibits the Enzymatic Activity of 17β-HSD3

The microsomal fraction derived from the homogenized rat testes was used to assess the enzymatic activity of 17β-HSD3. The enzymatic activity of 17β-HSD3 was determined in terms of the yield of T in the presence or absence of H10. The T yield ratio was 78.41% ± 7.41%, 51.86% ± 10.03%, and 45.14% ± 8.49% following treatment with H10 at doses of 10, 20, and 40 μM, respectively (**Figure 3E**), compared with those of the control group (*P* < 0.05). These results demonstrated that H10 inhibited the enzymatic activity of 17β-HSD3.

### H10 Reduced T Production and Had No Adverse Effects on the Male Reproductive System of Rats Following i.p. Administration for 7 Days

**Figure 4:**
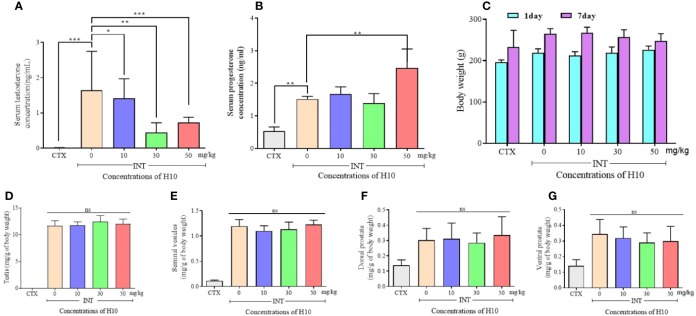
Numerical chart showing the serum levels of **(A)** T, and **(B)** P. **(C)** The levels and body weights, and weights of the **(D)** testes, **(E)** seminal vesicles, **(F)** dorsal prostate, and **(G)** ventral prostate of SD rats following the i.p. injection of H10 at doses of 0, 10, 30, and 50 mg/kg for 7 consecutive days. Following the i.p. administration of H10 to SD days for 7 consecutive days, the levels of T were significantly suppressed, although the serum levels of P slightly increased following the administration of H10 at a dose of 50 mg/kg. There were no significant differences in the body weights of the SD rats, along with the weights of the testes, seminal vesicle and prostate, seminal vesicle, and testes following H10 treatment in comparison with those of the normal SD rats. However, the index parameter of the castrated SD rats significantly decreased in comparison to those of the INT groups (n = 6, means ± sd, *P < 0.05, **P < 0.01, and ***P < 0.001 are considered significant *vs.* the INT H10 (0 mg/kg) group, ns indicates no significant difference *vs.* the INT H10 [0 mg/kg] group, *P* > 0.05).

The serum levels of T and P in the castrated group (CTX group) were much lower than those of the other groups, owing to the castration surgery. The other groups comprised the complete reproductive function (INT) groups. We observed that H10 lowered the levels of T in comparison to those of the group that received the solvent (**Figure 4A**). On the other hand, there was no significant difference in serum levels of P among the INT groups. However, H10 increased the serum levels of P at higher doses (**Figure 4B**). H10 had no effects on the body weights of the rats (**Figure 4C**), and on the weights of the testes (**Figure 4D**), seminal vesicle (**Figure 4E**), and prostate (**Figures 4F, G**), while the above index parameters of the castrated rats decreased significantly.

### H10 Inhibits the Growth of Tumor Xenografts Established From LNCaP Cells in Nude Mice *In Vivo*

**Figure 5:**
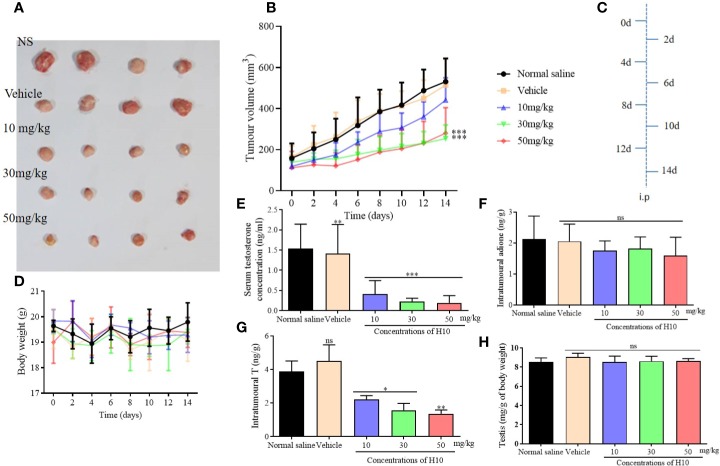
Antitumor effects of H10 at doses of 10, 30, and 50 mg/kg in LNCaP cells of the bearded nude mice model. **(A)** Diagrams depicting the tumor. **(B)** Tumor sizes. **(C)** Treatment schedule of the mice that were treated with drugs. **(D)** Body weights of the mice. T levels in the **(E)** serum and **(G)** tumor. **(F)** Intratumor levels of adione. **(H)** Testicular mass of each of the groups. The results demonstrated that treatment with H10 inhibited tumor growth, and the T levels in both the sera and tumor declined significantly. There was no significant difference in the body weights and testicular masses of the nude mice among the groups following the administration of H10 (n = 6, means ± sd, *P < 0.05, **P < 0.01, and ***P < 0.001 are considered significant *vs.* vehicle group, ns indicates no significant difference *vs.* vehicle group, *P* > 0.05.)

The tumor growth of male mice that received tumor xenografts from nude mice, was significantly inhibited following treatment with H10 on every alternate day, in comparison to that of the mice that received the solvent only (*P* < 0.05, **Figure 5A**). The increase in tumor volume was slowest following treatment with H10 at a dose of 50 mg/kg, being approximately 271 ± 46 mm^2^ at the end of the experiment, while the tumor volumes of the solvent group were 800–1,100 mm^2^. The *in vivo* tumor inhibitory effect of H10 was dose-dependent in the range between 10 and 50 mg/kg (**Figure 5B**). The body weights of the mice were relatively stable during the period of administration (**Figure 5D**), which indirectly indicated that the *in vivo* toxicity of H10 was relatively low.

The T levels in the sera and tumors of the H10 treatment groups were significantly lower than those of the normal saline group and the vehicle group (*P* < 0.05, **Figures 5E, G**). The T levels decreased as the dose of H10 was increased. However, H10 treatment did not alter the levels of adione and the weights of the testes (**Figures 5F, H**). As 17β-HSD3 is the key enzyme that mediates the transformation of adione to T, the changes in the levels of both adione and T results from the alterations in the enzymatic activity of 17β-HSD3 in adione-stimulated LNCaP proliferative tumors.

**Figure 6:**
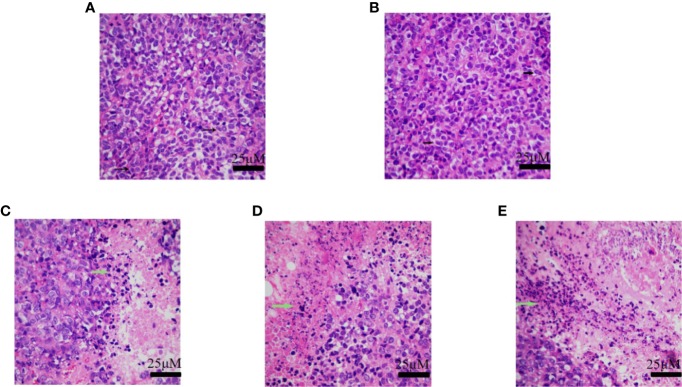
H&E staining of tumor tissue of the group receiving **(A)** normal saline, **(B)** vehicle, **(C)** 10 mg/kg of H10, **(D)** 30 mg/kg of H10, and **(E)** 50 mg/kg of H10. The arrows indicate the nuclei.

The tumor tissues of the normal saline group and the vehicle group showed obvious cellular atypia, comprising disordered cells having different morphological sizes, and showed the presence of meganuclei, along with binuclear, multinuclear, or heteronuclear nuclei, and a large nuclear to cytoplasmic ratio. The tumor tissues of the groups that received H10 showed the presence of large areas of tissue necrosis, including nuclear necrosis and nuclear disappearance. Compared to that of the normal mice, there were no significant differences among the different groups of mice with respect to the weights of the organs, as revealed by H&E staining (**Figure 6**). Analysis of the staining of the cellular proliferation marker, Ki67 and CD31-expression of the H10-treated mice, revealed that KI67 and CD31 was dose-dependently reduced (P < 0.001, **Figure S1**). However, a dose of 50 mg/kg significantly increased the expression of AR (P < 0.01, **Figures S2B, D**), but there was no significant difference in the expression of 17βHSD3 (P > 0.05, **Figures S2A, C**).

## Discussion

Compounds H1 to H12 differed with respect to the structures of their parent nuclei and the substituents (R) on the phenyl ring. Their structures were symmetrical, with two phenyl rings. The 17β-HSD3 inhibitory potential of H10, carrying chloride substituents, was better than that of the other analogs that did not possess the chloride substituents in their structures. The results of this study suggested the importance of the chloride substituent at this position in the phenyl ring over the other functional groups. In order to refine our compound selection strategy, the inhibitory potencies of the compounds were compared at lower concentrations of 0.25 to 1 μM (**Figure 2**). Previous studies have demonstrated that there are species-specific differences in the inhibitory potencies of compounds against this enzyme. As the expression of 17β-HSD3 in LC540 cells is negligible, the cells were transfected with 17β-HSD3, for constructing a cell line that stably expressed the 17β-HSD3 enzyme (LC540 [17βHSD3]) that was used in this study. However, the LC540 cells were transfected with rat 17β-HSD3 rather than human 17β-HSD3 in our study. Therefore, the aforementioned species-specific differences in the inhibitory potential of compounds in suppressing 17β-HSD3 mRNA expression requires further investigation. H10 strongly inhibited the T levels in LC540 (17β-HSD3) cells. Additionally, H10 downregulated the levels of T in the blood by inhibiting 17β-HSD3 in LNcap tumor of nude mice models *in vivo*. The results demonstrated that H10 was a potential molecule that can be developed as a treatment option for hormone-dependent prostate cancer.

In conclusion, the 17β-HSD3 inhibitory activities of a series of curcumin derivatives were investigated by studying the levels of T produced by LC540 (17β-HSD3) cells. In particular, H10 inhibited the enzymatic activity of 17β-HSD3, which subsequently decreased the levels of T. The mRNA and protein expression levels and the enzymatic activity of 17β-HSD3 were subsequently investigated. H10 reduced the production of T and had no adverse effects on the male reproductive system of SD rats following i.p. administration for 7 days. The results of these experiments indicated that H10 has 17β-HSD3 inhibitory activity. The hypothesis was validated by further studies on male mice harboring LNcap tumor xenografts. H10 can be used as a candidate 17β-HSD3 inhibitor for the treatment of hormone-dependent prostate cancer.

## Data Availability Statement

All datasets generated for this study are included in the article/**Supplementary Material**.

## Ethics Statement

The animal study was reviewed and approved by Animal Experimentation of Jinan University.

## Author Contributions

QX, Y-DH, and YY designed the study and developed the methodologies. YC, YW, WW, LX, YZ, SZ, and JT conducted the research. YC, YY, YW, LX, and SZ analyzed the data and provided the critical reagents. QX, YC, and YY prepared and revised the manuscript.

## Funding

This work was supported by the Major Scientific and Technological Special Project of the Administration of Ocean and Fisheries of Guangdong Province (Grant No. GDME-2018C013), Guangzhou Science and Technology Program key projects (Grant No. 201803010044).

## Conflict of Interest

The authors declare that the research was conducted in the absence of any commercial or financial relationships that could be construed as a potential conflict of interest.
