# Supplementary material for: The Curcumin Derivative, H10, Suppresses Hormone-Dependent Prostate Cancer by Inhibiting 17β-Hydroxysteroid Dehydrogenase Type 3
Source: Front Pharmacol. 2020 May 8;11:637. doi: 10.3389/fphar.2020.00637 (PMC7227374; doi:10.3389/fphar.2020.00637)
Supplement: Supplementary file 1 [file DataSheet_1.docx]

Supplementary Material

# Supplementary Data

The tumors were fixed in formalin. The paraffin-embedded tissue blocks were dewaxed, rehydrated, and blocked for studying the endogenous peroxidase activity. Antigen retrieval was performed in a sodium citrate buffer (0.01 mol/L, pH 6.0) in a microwave oven at 1,000 W for 10 minutes. Nonspecific antibody binding was blocked by incubating with 5% bovine serum albumin in PBS for 30 minutes at room temperature. The slides were then individually incubated with anti-Ki-67 (at 1:100; Affinity), anti-CD-31 (at 1:100; Affinity), anti-AR (at 1:100; Affinity), and anti-17β-HSD3 (at 1:100; Affinity) antibodies at 4°C overnight. After rinsing with PBS, the slides were washed and incubated with rabbit secondary antibodies for 40 minutes. After washing four times with PBS, the sections were treated with DAB and hematoxylin for staining and re-dying the nucleus, respectively. The sections were finally dehydrated and sealed with Permount^TM^ Mounting Medium for microscopic observation (Olympus IX71, Tokyo, Japan).

Analysis of the staining of the cellular proliferation marker, Ki67, in the tumor tissues, and CD31 staining in the endothelial cells of the tumors of the H10-treated mice, revealed that KI67 and CD31-expression effect of H10 was dose-dependently reduced in the range between 10 mg/kg and 50 mg/kg (P < 0.001, Figure S1.A and C). However, treatment with H10 at a dose of 50 mg/kg significantly increased the expression of AR (*P* < 0.01, Figure S2.B and D), but there was no significant difference in the expression of 17βHSD3 (*P* > 0.05, Figure S2.A and C).

# Supplementary Figures and Tables

## Supplementary Figures

**
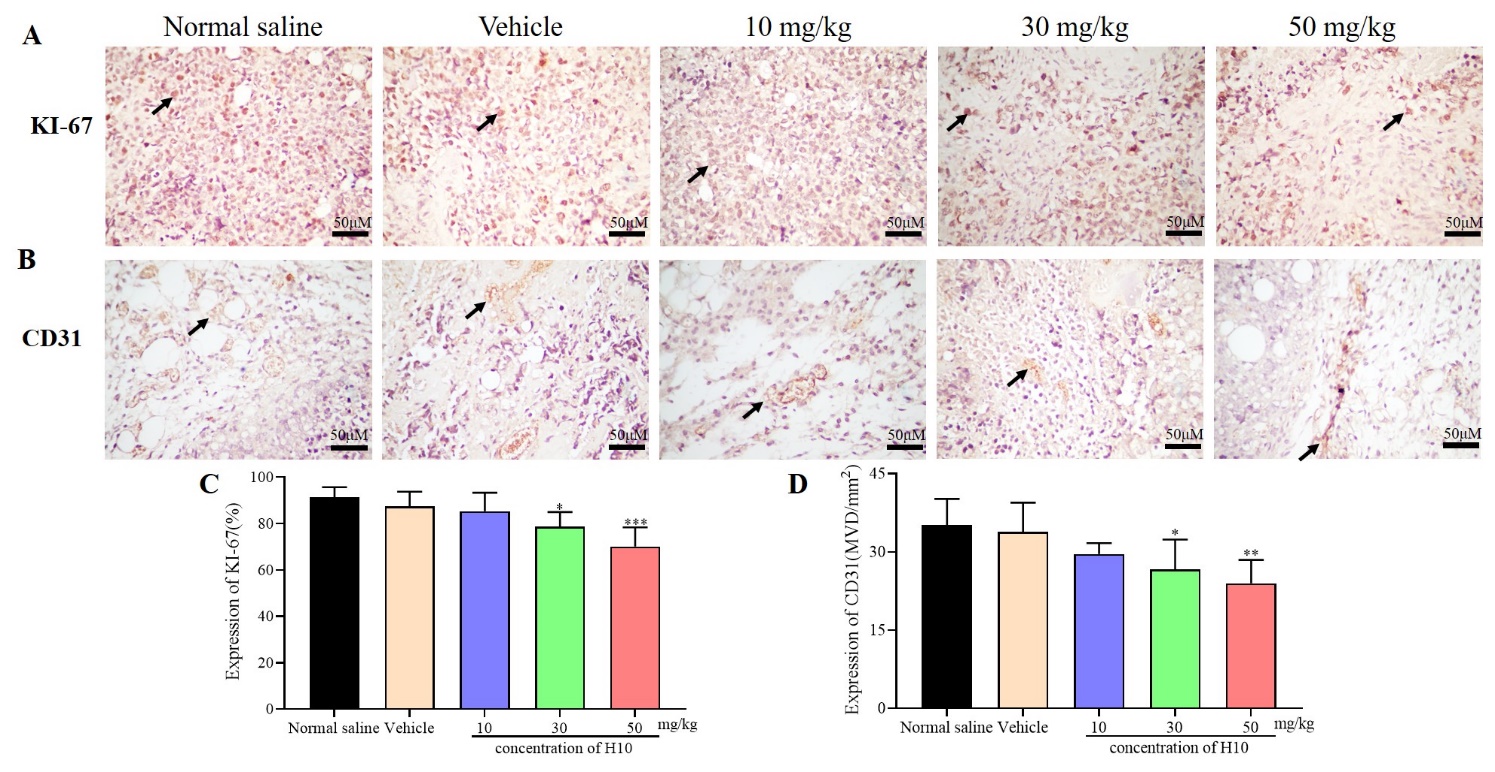
**

**Supplementary Figure 1.** Immunohistochemical examination of KI-67 and CD-31 expression in tumor tissue. **(A)** Immunohistochemical labeling of KI-67-positive cells. Arrows indicate KI-67 expression. **(C)** Quantitative analysis of KI-67-expressing cells using ImageJ software. **(B)** Immunohistochemical labeling of CD-31-positive cells. Arrows indicate CD31 expression. **(D)** Quantitative analysis of CD-31-expressing cells using ImageJ software. n = 6, means ± sd, *P < 0.05, **P < 0.01, ***P < 0.001, vs control group, ns means no significant difference vs. control, P >0.05.


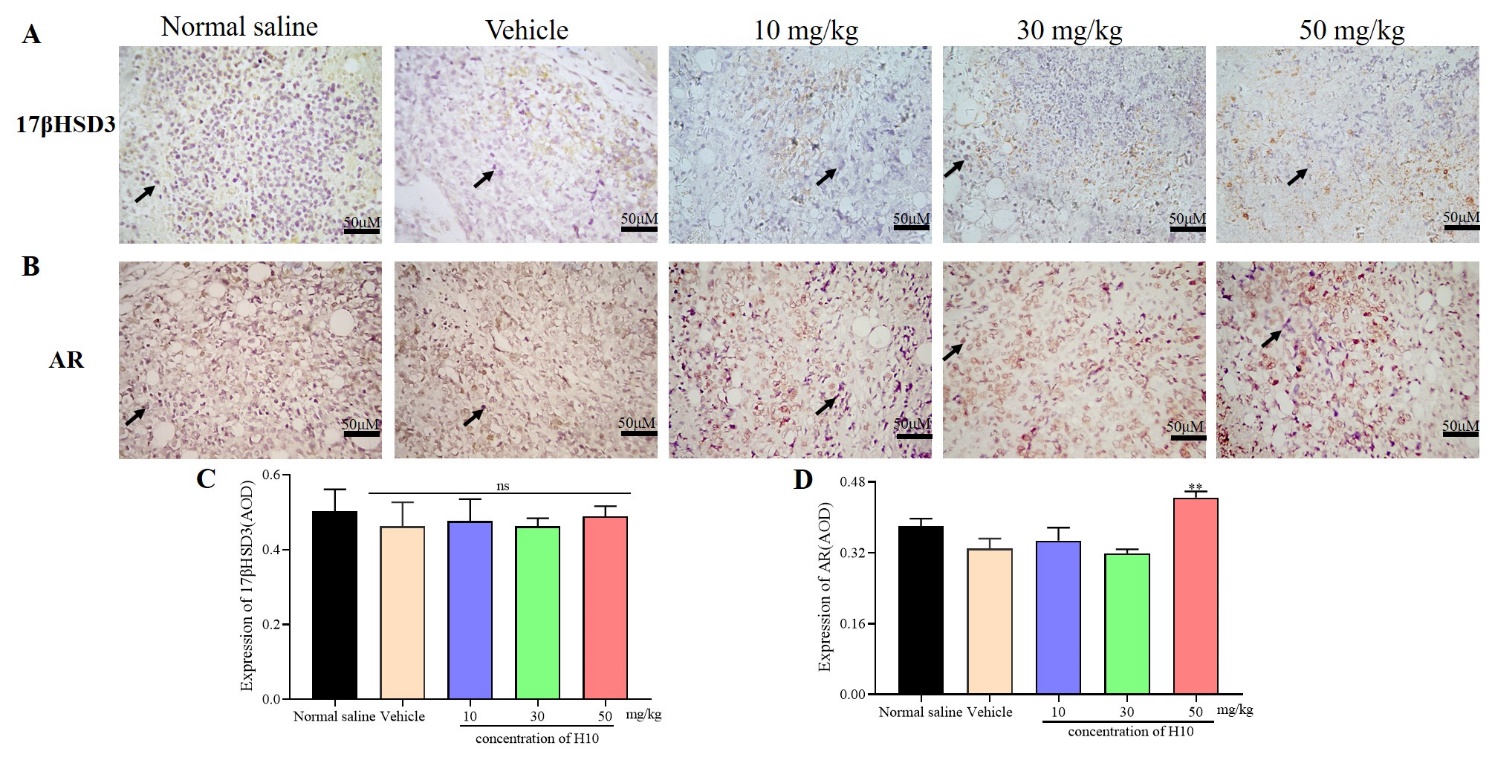


**Supplementary Figure 2.** Immunohistochemical examination of 17βHSD3 and AR expression in tumor tissue. **(A)** Immunohistochemical labeling of 17βHSD3-positive cells. Arrows indicate 17βHSD3 expression. **(C)** Quantitative analysis of 17βHSD3-expressing cells using ImageJ software. **(B)** Immunohistochemical labeling of AR-positive cells. Arrows indicate AR expression. **(D)** Quantitative analysis of AR-expressing cells using ImageJ software. n = 6, means ± sd, **P < 0.01 vs control group, ns means no significant difference vs. control, P >0.05.
